# Characterization of merozoite-specific thrombospondin-related anonymous protein (MTRAP) in *Plasmodium vivax* and *P. knowlesi* parasites

**DOI:** 10.3389/fcimb.2024.1354880

**Published:** 2024-02-23

**Authors:** Nguyen Sy Thau, Tuyet-Kha Nguyen, Nguyen Van Truong, Thi-Thanh Hang Chu, Sung-Hun Na, Robert W. Moon, Yee Ling Lau, Myat Htut Nyunt, Won-Sun Park, Wan-Joo Chun, Feng Lu, Seong-Kyun Lee, Jin-Hee Han, Eun-Taek Han

**Affiliations:** ^1^ Department of Medical Environmental Biology and Tropical Medicine, Kangwon National University School of Medicine, Chuncheon, Gangwon-do, Republic of Korea; ^2^ Department of Obstetrics and Gynecology, Kangwon National University School of Medicine, Chuncheon, Gangwon-d, Republic of Korea; ^3^ Department of Infection Biology, Faculty of Infectious and Tropical Diseases, London School of Hygiene and Tropical Medicine, London, United Kingdom; ^4^ Department of Parasitology, Faculty of Medicine, Universiti Malaya, Kuala Lumpur, Malaysia; ^5^ Department of Medical Research, Yangon, Myanmar; ^6^ Department of Physiology, School of Medicine, Kangwon National University, Chuncheon, Gangwon-do, Republic of Korea; ^7^ Department of Pharmacology, School of Medicine, Kangwon National University, Chuncheon, Gangwon-do, Republic of Korea; ^8^ Department of Pathogen Biology and Immunology, School of Medicine, Yangzhou University, Yangzhou, Jiangsu, China

**Keywords:** malaria, Plasmodium vivax, Plasmodium knowlesi, MTRAP, invasion, cross-species immune responses

## Abstract

*Plasmodium vivax*, the most widespread human malaria parasite, and *P. knowlesi*, an emerging *Plasmodium* that infects humans, are the phylogenetically closest malarial species that infect humans, which may induce cross-species reactivity across most co-endemic areas in Southeast Asia. The thrombospondin-related anonymous protein (TRAP) family is indispensable for motility and host cell invasion in the growth and development of *Plasmodium* parasites. The merozoite-specific TRAP (MTRAP), expressed in blood-stage merozoites, is supposed to be essential for human erythrocyte invasion. We aimed to characterize MTRAPs in blood-stage *P. vivax* and *P. knowlesi* parasites and ascertain their cross-species immunoreactivity. Recombinant *P. vivax* and *P. knowlesi* MTRAPs of full-length ectodomains were expressed in a mammalian expression system. The MTRAP-specific immunoglobulin G, obtained from immune animals, was used in an immunofluorescence assay for subcellular localization and invasion inhibitory activity in blood-stage parasites was determined. The cross-species humoral immune responses were analyzed in the sera of patients with *P. vivax* or *P. knowlesi* infections. The MTRAPs of *P. vivax* (PvMTRAP) and *P. knowlesi* (PkMTRAP) were localized on the rhoptry body of merozoites in blood-stage parasites. Both anti-PvMTRAP and anti-PkMTRAP antibodies inhibited erythrocyte invasion of blood-stage *P. knowlesi* parasites. The humoral immune response to PvMTRAP showed high immunogenicity, longevity, and cross-species immunoreactivity with *P. knowlesi*. MTRAPs are promising candidates for development of vaccines and therapeutics against vivax and knowlesi malaria.

## 1 Introduction

Worldwide, with an estimated 247 million cases and 619,000 deaths annually (WHO, 2022), malaria, which is caused by the *Plasmodium*, is a leading health burden. After entering human circulation, *Plasmodium* sporozoites invade liver cells to form liver merozoites, which subsequently enter red blood cells (RBCs) for the intraerythrocytic phase of their lifecycle (Cowman and Crabb, 2006). Except for a small proportion of parasitized erythrocytes that differentiate into gametocytes for subsequent transmission, most follow the asexual cycle that causes the major clinical symptoms of malaria (Baker, 2010). Within minutes after eruption from ruptured schizonts, daughter merozoites invade naïve erythrocytes through a series of molecular events: initial attachment, re-orientation, junction formation, and erythrocyte penetration by the parasite (Koch and Baum, 2016). The precise mechanism of erythrocyte invasion is complicated and has been more informatively studied in *P. falciparum* than in other *Plasmodium* human parasites.

Of the five human malaria parasites, *P. vivax* has the widest prevalence worldwide and is predominant in sub-Saharan Africa (Howes et al., 2016). With its unique biology, such as early inducement of gametocytes, restriction of invading reticulocytes, which causes low parasitemia that challenges clinical and paraclinical detection, and the ability to trigger hypnozoite-induced relapses, *P. vivax* constitutes a key target in the last global battle against malaria (Adams and Mueller, 2017; Bantuchai et al., 2022; Moreno et al., 2022). Although the involvement of some *P. vivax*-expressed proteins, such as *P. vivax* Duffy binding protein (PvDBP) (Kar and Sinha, 2022), and most recently *P. vivax* reticulocyte-binding protein 1a and 1b (PvRBP1a and 1b) (Han et al., 2016), 2a (PvRBP2a) (Malleret et al., 2021), and 2b (PvRBP2b) (Gruszczyk et al., 2018) in reticulocyte invasion are well documented, the mechanism underlying this process remains largely unknown (Molina-Franky et al., 2022). The recent increase in human malaria cases due to a zoonotic malaria parasite, *P. knowlesi*, with the highest prevalence in Southeast Asia (Jeyaprakasam et al., 2020), has revealed that the pathogen is able to induce severe, fatal disease. Furthermore, *P. knowlesi*-induced severe malaria occurs in 6–9% of symptomatic cases, with relatively low parasitemia (Grigg et al., 2018). In some regions where other parasites causing human malaria have been eliminated, the prevalence of *P. knowlesi* infection has dramatically increased, which makes it a predominant, high-priority target for malaria-control programs (Cooper et al., 2020). The identification of a cross-species immune response between *P. vivax* and *P. knowlesi* is in line with the fact that these parasites are not only phylogenetically close but also co-endemic (Longley et al., 2022). As *P. knowlesi*, but not *P. vivax*, can be culture *in vitro* for prolonged periods with human erythrocytes (Moon et al., 2013), *P. knowlesi* can be used as an model for research on both *P. knowlesi* and *P. vivax* (Muh et al., 2018a; Han et al., 2019; Verzier et al., 2019; Muh et al., 2020; Ndegwa et al., 2021).

The thrombospondin-related anonymous protein (TRAP) is a conserved superfamily encompasses vector-borne apicomplexan parasites, including *Plasmodium* spp. and *Babesia* spp (Morahan et al., 2009; Paoletta and Wilkowsky, 2022). *Plasmodium falciparum* TRAP constitutes the first-described sporozoite micronemal protein in the TRAP-family proteins (Robson et al., 1988), and although *Plasmodium* TRAP family members share a conserved structure related to parasite motility and host-cell invasion, they also present stage-specific features (Morahan et al., 2009). In *Plasmodium* biology, the TRAP family comprises both TRAP and TRAP-related protein (TREP), which are expressed on sporozoites (Robson et al., 1995; Combe et al., 2009; Steinbuechel and Matuschewski, 2009), whereas the circumsporozoite- and TRAP-related protein (CTRP) are expressed on ookinetes (Dessens et al., 1999). The TRAP-like protein (TLP) is present on both sporozoites and merozoites (Heiss et al., 2008; Moreira et al., 2008), and merozoite-specific TRAP (MTRAP) is found on merozoites (Baum et al., 2006). The MTRAP initially evaluated in *P. falciparum* (PfMTRAP) was subsequently studied extensively in asexual (Baum et al., 2006; Bartholdson et al., 2012; Uchime et al., 2012) and sexual blood-stage parasites (Bargieri et al., 2016). MTRAP is expressed in the middle-to-late stage of asexual intraerythrocytic parasites, binds to erythrocytes using its thrombospondin repeat (TSR) domains to interact with erythrocytic Semaphorin-7A, and is vital for the egress of the sexual stage, which may indicate the function of MTRAP in parasite motility (Baum et al., 2006; Bartholdson et al., 2012; Uchime et al., 2012; Bargieri et al., 2016). A recent report indicated that *P. vivax* MTRAP specifically interacts with human CD36 (Nguyen et al., 2023). Nevertheless, the protein features and pathophysiological activity of PvMTRAP and its homolog in *P. knowlesi* have not been unraveled yet. In this study, we aimed to characterize the MTRAPs of *P. vivax* and *P. knowlesi* parasites by assessing their subcellular localization, functional activity during erythrocyte invasion, and the humoral immune response in infected patients from *P. vivax* and *P. knowlesi* endemic areas.

## 2 Materials and methods

### 2.1 Sera of patients with *P. vivax*- and *P. knowlesi* infections

Blood samples of patients with *vivax* and *knowlesi* malaria were collected from three endemic areas: the Republic of Korea (ROK), Myanmar, and Malaysia. To evaluate the longevity of immune response, blood samples from patients with vivax malaria were collected from Shwegyin Township in Myanmar on the day of the patient’s visit (D0, acute infection), day 28 after proper treatment (D28, sub-acute) (**Supplementary Table S1**), and 1 year later, without reinfection (1 y). According to the National Malaria Treatment Guidelines, all participants received the appropriate treatment. This study was conducted concurrently with the therapeutic efficacy study of chloroquine in uncomplicated vivax malaria, using the template protocol provided by the WHO. A history of no malarial episodes (except for the relevant ones for D28 and 1 y samples) and current malarial infection status were confirmed from the data in the patient record files, microscopy, and species-specific polymerase chain reaction (PCR). For the negative control, blood samples of healthy individuals from non-endemic areas of the ROK were collected after microscopy- and PCR-based confirmation of malaria-free status. Sera were isolated from whole blood samples and stored at -80°C until a protein array was performed. All experiments complied with relevant guidelines and regulations, and all protocols involving human samples were approved by the Institutional Ethical Committee of the Department of Medical Research, Myanmar (Approval number 49/Ethics-2014), the Kangwon National University Hospital Ethical Committee (IRB No. 2014-08-008-002), University of Malaysia Medical Ethics Committee (Ref No. 817.18), and the Medical Research Ethics Committee (MREC), Ministry of Health, Malaysia (NMRR-16-2840-33769). Informed consent was obtained from all participants.

### 2.2 Bioinformatics analysis

In this study, sequences of MTRAP proteins of divergent *Plasmodium* species were retrieved from PlasmoDB (www.plasmodb.org), which included *P. vivax* (PVX_111290), *P. knowlesi* (PKNH_0613400), *P. falciparum* (PF3D7_1028700.1), *P. ovale curtisi* (PocGH01_06021700.1), and *P. cynomolgi* (PcyM_0617400-t36_1); *P. malariae* (XP_028862173.1) was obtained from GenBank. Sequence alignment was performed by the CLUSTAL-W program in MegAlign Lasergene ver. 7.0 (DNASTAR, Madison, WI). The primary structures of PvMTRAP and PkMTRAP were analyzed using the Simple Modular Architecture Research Tool (http://smart.emblheidelberg.de/). To obtain the predicted three-dimensional structures of PvMTRAP and PkMTRAP, the protein sequences were submitted to SWISS-MODEL (Biasini et al., 2014) and AlphaFold2 webserver (Jumper et al., 2021); the selected structures were then visualized and analyzed by UCSF Chimera (Huang et al., 2014).

### 2.3 Recombinant protein expression and purification

Recombinant PvMTRAP and PkMTRAP were produced using a mammalian HEK293E cell-expression system as described elsewhere (Patel et al., 2022). For plasmid preparation, GeneArt (https://www.thermofisher.com/order/geneartgenes/projectmgmt) was used to codon-optimize a cDNA sequence encoding the full-length ectodomain of PvMTRAP and PkMTRAP for expression in mammalian cells, and was followed by chemical synthesis with a Twist Bioscience tool (South San Francisco, CA, USA). The cDNAs were cloned into a pTT5 vector containing a leader sequence of mouse variable-k-light chain (mIgGk) as an exogenous signal peptide, and 8× His C-terminal tags were then transfected into HEK cells for protein production. In accordance with the manufacturer’s protocol, the His-tagged proteins were purified using Ni-NTA agarose (QIAGEN, Hilden, Germany) and Poly-Prep® Chromatography column (Bio-Rad, Hercules, CA, USA).

### 2.4 Animal antibody production and immunoglobulin purification

For producing polyclonal antibodies against PvMTRAP and PkMTRAP, we used 6- to 8-week-old female BALB/c mice (Daehan Biolink Co., Eumsung, ROK). Twenty microliters of purified recombinant proteins mixed with complete Freund’s Adjuvant (Sigma-Aldrich, St. Louis, MO) was injected intraperitoneally into each mouse. At weeks 3 and 5 following the initial immunization, a mixture of the same quantities of antigens with incomplete Freund’s Adjuvant (Sigma-Aldrich) was administered as booster doses. Mouse sera were collected 2 weeks after the final boost. For producing rabbit antibodies against PvMTRAP and PkMTRAP, 250 μg protein mixed with complete Freund’s Adjuvant was administered subcutaneously to Japanese white rabbits. Two booster immunizations with 250 μg protein mixed with incomplete Freund’s Adjuvant were given every 3 weeks, and rabbit sera were obtained 2 weeks after the final injection. In accordance with the manufacturer’s instructions, IgG purification from rabbit sera was performed using a protein G HP column (GE Healthcare Life Sciences, Buckinghamshire, UK). The elution fractions were then subjected to buffer exchange for incomplete RPMI1640 (Invitrogen, Carlsbad, CA, USA) and concentrated at 30 kDa using centrifugal devices (Millipore, Billerica, MA, USA) for downstream assay.

### 2.5 SDS-PAGE and Western blotting

Protein lysates from *P. knowlesi* A1-H.1 schizont-stage parasites were obtained as previously described (Muh et al., 2018a). After treatment with a reducing agent to induce a reducing condition, *P. knowlesi* schizont lysates or His-tagged recombinant proteins were separated by 13% SDS-PAGE gel. A gel for SDS-PAGE analysis was stained with Coomassie brilliant blue (Sigma-Aldrich). Concurrently, another gel was used for protein blotted onto PVDF membranes (Millipore) and blocked with 5% skim milk at room temperature (RT) for 1 h. The membrane containing His-tagged protein was then incubated with either mouse anti-penta-His antibody (1:2,000) or animal immune sera (1:1,000) against PvMTRAP or PkMTRAP, followed by incubation with a secondary goat anti-mouse IRDye^®^ 800 (1:10,000) or goat anti-rabbit IRDye^®^ 680 (1:20,000) antibody. Data analysis was performed using an Odyssey infrared imaging system and software (LI-COR^®^ Bioscience).

### 2.6 Protein array

Amine-coated slides were prepared and used as described elsewhere (Chen et al., 2010). To evaluate the humoral immune response to PvMTRAP and PkMTRAP, 1 µL of each purified recombinant protein was spotted onto duplicate wells of the array at 100 ng/μL. After blocking with 5% bovine serum albumin in phosphate-buffered saline (PBS) with 0.1% Tween 20, the slides were incubated with serum samples from patients with *vivax* or *knowlesi* malaria or unexposed healthy individuals (1:25 dilution) at 37 °C for 1 h. Non-binding human IgG was washed from the slides, whereas bound IgG were labeled with Alexa Fluor 546 goat anti-human IgG antibody (10 ng/µL; Invitrogen) for detection using a fluorescence scanner (InnoScan, Carbonne, France). A cutoff value was established at the mean fluorescence intensity (MFI) plus two standard deviations (SD) of healthy individuals, which was then used to normalize individual MFI (Muh et al., 2017).

### 2.7 Indirect immunofluorescence assay

*Plasmodium knowlesi* A1-H.1 parasites were fixed with a mixture of 4% paraformaldehyde and 0.075% glutaraldehyde and used for further immunostaining procedures. The fixed parasites were permeabilized with 0.1% Triton X-100 in PBS for 30 min at RT. The slides were then co-stained with mouse serum against either PvMTRAP or PkMTRAP as well as each rabbit serum against *P. knowlesi* merozoite surface protein 1–19 (PkMSP1–19) (Lee et al., 2023), *P. knowlesi* Duffy binding protein (PkDBP) (Hart et al., 2023), *P. vivax* rhoptry neck protein (PvRON2) (Arévalo-Pinzón et al., 2011), and *P. vivax* rhoptry-associated membrane antigen proteins (PvRAMA) (Lu et al., 2014) as subcellular localization makers for merozoite surface, microneme, rhoptry neck, and rhoptry body, respectively. After washing, the IgG binding was probed with Alexa Fluor 568-conjugated anti-mouse IgG (H+L) and Alexa Flour 488-conjugated anti-rabbit IgG (H+L) antibody (1:500, Invitrogen), whereas nuclei were stained with 4′,6-diaminidino-2-phenylindole (DAPI) (1:1000, Invitrogen). The slides were mounted with ProLong Gold antifade reagent (Invitrogen) and visualized under oil immersion using a Nikon C2 confocal microscope (Nikon Instrument Inc., City, NY, USA) equipped with a 60× oil objective. The acquired images were prepared using ImageJ.

### 2.8 Enrichment of *P. knowlesi* A1-H.1 schizont using Percoll gradient

The *P. knowlesi* A1-H.1 strain was cultured with human erythrocytes in RPMI 1640-based medium (Invitrogen/Life Technologies, Grand Island, NY, USA), as described previously (Moon et al., 2013). The Percoll solutions were freshly prepared by mixing nine parts of Percoll density gradient media (Cytiva, Marlborough, MA, USA) with one part 10× PBS. Subsequently, this stock solution was diluted with 1× PBS to the desired concentrations. The parasite culture with predominant schizont stage was pelleted and carefully overlayed on the 40%/70% Percoll gradient before centrifugation at 900 ×g for 12 min without brake. The schizonts collected from the lower interphase were washed three times with RPMI prior to use in downstream experiments. A schizont enrichment assay using magnetic-activated cell sorting (MACS) (Miltenyi Biotec, Bergisch Gladbach, Germany) with an LD column (Meltenyi Biotec), well-established for *P. knowlesi* A1-H.1, was performed in parallel to Percoll to compare the proliferation feature of enriched schizonts (Amir et al., 2016).

### 2.9 Invasion inhibition assay

The invasion inhibition assay was performed as previously described (Muh et al., 2018b). Briefly, purified schizonts of *P. knowlesi* A1-H.1 were sub-cultured in a 96-well plate at 2% hematocrit and 1.0–1.5% parasitemia. Purified rabbit IgG against PvMTRAP or PkMTRAP at serial concentrations were added to duplicate wells. Cultures without antibodies or with Duffy antigen receptor for chemokines (DARC) monoclonal antibody (2C3, Absolute antibody, Oxford, UK) at 25 µg/mL served as controls for normal erythrocyte invasion and antibody-mediated invasion inhibition, respectively. The sub-culture was maintained in a humidified chamber under mixed gas (90% N_2_, 5% CO_2_, 5% O_2_) at 37°C until newly invaded ring-stage parasites were detected on Giemsa staining (approximately 10 h). Parasites were fixed with 0.05% glutaraldehyde in PBS, then stained with SYBR Green (1:10,000; Invitrogen). A total of 200,000 events were counted per sample using a FACS Accuri™ C6 Flow Cytometer (Becton-Dickinson Co., Mansfield, MA, USA). Data were analyzed using FlowJo (Treestar, Ashland, OR, USA).

### 2.10 Statistical analysis

The data were analyzed using GraphPad Prism (GraphPad Software, San Diego, CA, USA) and Microsoft Excel 2018 (Microsoft, Redmond, WA, USA). During the comparison of experimentally measured values across groups, the Student’s *t*- and Mann–Whitney *U*-tests were used. Spearman analysis was utilized to examine the correlation of nonparametric data; *p*<0.05 indicated significant differences.

## 3 Results

### 3.1 Schematic primary structure of PvMTRAP and PkMTRAP

**Figure 1:**
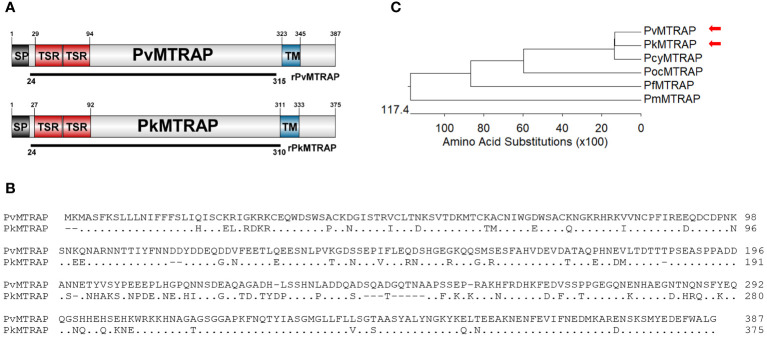
Schematic of protein primary structure and sequence analysis of PvMTRAP and PkMTRAP proteins. **(A)** Schematic depiction of PvMTRAP and PkMTRAP. Sequences for the expression of recombinant proteins rPvMTRAP (residues 24–315) and rPkMTRAP (residues 24–311) are indicated as black lines. The signal peptide (SP), transmembrane domain (TM), and thrombospodin repeat (TSR) are represented. **(B)** Clustal alignment of full-length PvMTRAP and PkMTRAP amino acid (aa) sequences; the aa of PkMTRAP, which are identical to those of PvMTRAP, are represented as dots. The hyphen indicates the alignment-induced gaps within PkMTRAP. **(C)** The phylogenetic tree indicates the relationships among MTRAP sequences of various human-infected malaria parasites. Red arrowheads indicate the positions of PvMTRAP and PkMTRAP.

The PvMTRAP and PkMTRAP proteins consisted of 387 and 375 amino acids, respectively, and both were predicted as transmembrane type 1 proteins with a signal peptide followed by two TSR domains at the N-terminal (**Figure 1A**). The repeated sequence of “WxxWxxC~G~R~C” (“x” could be substituted by any amino acid) was largely conserved among TSR domains of MTRAP from human-infected malarial parasites and human thrombospondin-1 protein (**Supplementary Figure S1A**). PvMTRAP and PkMTRAP sequences shared 77.7% identity, and thus exhibited the closest phylogenetic relationship among malarial parasites that infect humans (**Figures 1B, C**; **Supplementary Figure S1B**).

### 3.2 Three-dimensional structure analysis of the TSR domains of PvMTRAP and PkMTRAP

**Figure 2:**
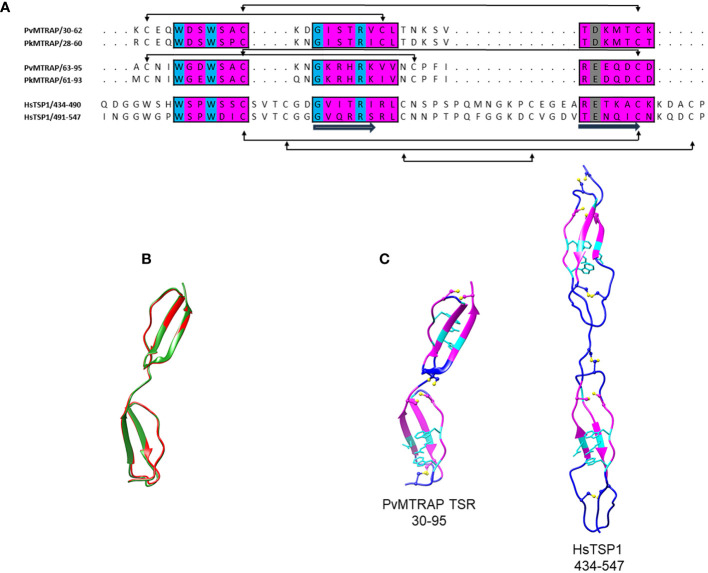
Three-dimensional structural analysis of PvMTRAP and PkMTRAP. **(A)** Sequence alignment of the TSR domains of PvMTRAP (residues 30–95), PkMTRAP (residues 28–93), and human thrombospondin 1 (HsTSP1; residues 434–547). Three regions of each TSR domain that are predicted to form its stacked core are highlighted in magenta boxes, with conserved aa in cyan. The aspartic and glutamic acid residues predicted to be involved in hydrogen bond formation with the conserved central stacked arginine are indicated in gray. The secondary structure and disulfide bridges are shown in the alignment. **(B)** A structural superposition of PvMTRAP TSR (in red) and PkMTRAP TSR (in green). **(C)** Predicted 3D structure of PvMTRAP-TSRs and the crystal structure of HsTSP-1-TSRs. Disulfide linkages are highlighted with sticks and bones in yellow. Regions predicted to align between the MTRAP-TSRs and the HsTSP-1-TSRs are indicated in magenta, with conserved residues forming the stacked core, and hydrogen bonds with arginine in the TSR domains shown as cyan sticks.

Given the unsuccessful attempt to find an experimentally determined template for PvMTRAP and PkMTRAP, the full protein sequences were submitted to an AlphaFold 2 webserver for predicting their 3D structures. Only TSR domains of MTRAP proteins exhibited the sufficient per-residue confidence score (high and very high, per-residue model confidence score >70) and were selected for further comparison with those of human thrombospondin-1 (HsTSP-1; PDB identification 1LSL) (Uchime et al., 2012). The TSR domains of PvMTRAP and PkMTRAP shared 81.8% sequence identity, and their 3D structures were predicted to be extensively overlapped (**Figures 2A, B**). As predicted, each TSR domain of PvMTRAP and PkMTRAP folded into a long, thin, antiparallel, three-stranded domain that was similar to that of HsTSP-1 (Tan et al., 2002); however, significantly more dense structures were noticed in MTRAP-TSR domains (**Figure 2C**), which likely resulted from the prediction that a single TSR domain of MTRAP was approximately half the length of the HsTSP-1 TSR domain (~27.76 Å compared to ~55 Å, respectively), and linkers between the two TSR domains of MTRAPs had eight fewer amino acids than that of HsTSP-1 (**Figures 2A, C**). The PvMTRAP/PkMTRAP TSR domains included four disulfide bridges, which is less than the six found in HsTSP-1 (**Figures 2A, C**). The disulfide bonds between a cysteine at the N terminal of A-loop and a cysteine at the strand B (the first TSR domain) or in the B-C loop (the second TSR domain) were considered specific for MTRAP as it was not found in HsTSP-1 (**Figures 2A, C**) or TRAP-TSR domains (Tan et al., 2002).

### 3.3 Production of purified recombinant proteins and specific IgG from immune animals

**Figure 3:**
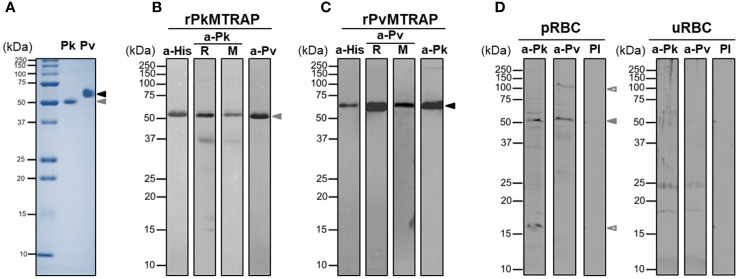
SDS-PAGE and Western blot analysis of PvMTRAP and PkMTRAP. **(A)** Recombinant PkMTRAP (Pk) and PvMTRAP (Pv) are expressed and purified before resolution in SDS-PAGE. **(B)** Purified PkMTRAP (rPkMTRAP) is specifically detected by anti-His antibody (a-His), anti-PkMTRAP IgG (a-Pk) from immunized rabbit (R) and mouse (M), and anti-PvMTRAP IgG (a-Pv). **(C)** Purified PvMTRAP (rPvMTRAP) is specifically recognized by a-His, a-Pv from immunized rabbit (R) and mouse (M), and a-Pk. **(D)** Lysate protein from *P. knowlesi* A1-H.1 schizont (pRBC) or uninfected human erythrocyte (uRBC) is probed with anti-PkMTRAP (a-Pk), anti-PvMTRAP (a-Pv), and pre-immune IgG (PI). Solid black and brown arrowheads represent PvMTRAP and PkMTRAP target bands, respectively; non-filled brown arrowheads indicate possibly processed fragments of native PkMTRAP.

The full-length ectodomains of PvMTRAP and PkMTRAP proteins were expressed and purified as single bands of approximately 57.8 and 51.5 kDa, respectively (**Figure 3A**). Western blot analysis using anti-His antibody confirmed the migration pattern of recombinant PvMTRAP and PkMTRAP observed by SDS-PAGE (**Figures 3B, C**, lane a-His). At 32.4 and 32.5 kDa for PvMTRAP and PkMTRAP, respectively, the product weight exceeded the predicted molecular weight; this may have resulted from post-translational modifications and/or the structure of MTRAP proteins, as the same phenomenon was found in PfMTRAP (Baum et al., 2006). Antibodies from rabbits and mice immunized with recombinant PvMTRAP and PkMTRAP were able to recognize the respective antigens similarly to an anti-His antibody (**Figures 3B, C**, lane R and M); furthermore, anti-PvMTRAP IgG cross-reacted with recombinant PkMTRAP (**Figure 3B**, lane a-Pv) and vice versa (**Figure 3C**, lane a-Pk). Interestingly, antibodies against PkMTRAP and PvMTRAP explicitly bound to the native protein from *P. knowlesi* schizont lysate (approximately 51.0 kDa; **Figure 3D**), giving an additional indication of cross-species immunoreactivity between PkMTRAP and PvMTRAP. The extra bands detected by anti-MTRAP IgGs may represent processing fragments of MTRAP proteins during parasitic development (**Figure 3D**).

### 3.4 Subcellular localization of PvMTRAP and PkMTRAP proteins

**Figure 4:**
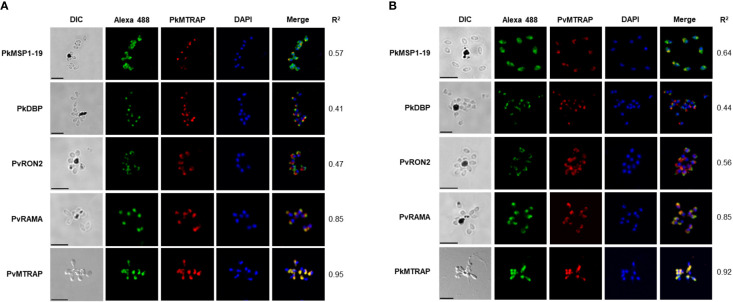
Subcellular localization of PkMTRAP **(A)** and PvMTRAP **(B)**. Schizonts are dual stained with mouse anti-PvMTRAP/PkMTRAP antibodies (in red) and rabbit antibodies specifically against PkDBP, PkMSP1-19, PvRAMA, PvRON2, and PkMTRAP/PvMTRAP (in green). The 4’,6-diaminidino-2-phenylindole (DAPI) is used to stain nuclei (in blue). Pearson’s *r* ^2^ represents the extent of colocalization between the red signal (target protein) and green signal (subcellular markers) in each stained schizont. DIC, differential interference contrast; bar scale: 5 μm.

The fluorescence signal of the antibody against PkMTRAP presents as a punctured pattern, indicating that PkMTRAP localizes to an apical part of the merozoite (**Figure 4A**, in red). Unlike PfMTRAP (Baum et al., 2006), PkMTRAP does not reside in the microneme, and this inference is drawn from the distinct signal pattern observed with the anti-PkMTRAP antibody, which differs from that of the anti-PkDBPα antibody, the latter serving as a marker for *P. knowlesi* merozoite micronemes (Hart et al., 2023).Antibodies to PvRAMA, a *P. vivax* rhoptry body protein, and PvRON2, a *P. vivax* rhoptry neck protein, were both able to recognize their orthologous in rhoptry of *P. knowlesi* merozoite (Muh et al., 2018a), suggesting that they could be used as subcellular localization makers for rhoptry region of *P. knowlesi*. Given the lack of evidential subcellular markers for *P. knowlesi* rhoptries and our attempt to further localize PkMTRAP between the bulb and neck region of merozoite rhoptry, antibodies against PkMTRAP were co-stained with antibodies against PvRAMA and PvRON2. The signals from PkMTRAP mostly overlapped with those from PvRAMA (*r* ^2 =^ 0.85), but not PvRON2 (*r* ^2 =^ 0.47), (**Figure 4A**) revealing that (i) *P. knowlesi* orthologous of PvRAMA and PvRON2 might localize to distinct parts of the rhoptry, and (ii) the rhoptry body was likely the localization of PkMTRAP.

Extensively overlapping signals between rabbit antisera against PvMTRAP and mouse antisera against PkMTRAP (*r* ^2 =^ 0.95, **Figure 4A**, bottom line) and in reverse orientation (*r* ^2 =^ 0.92, **Figure 4B**, bottom line) indicated that anti-PvMTRAP could cross-react with native PkMTRAP antigens. Moreover, replacing anti-PkMTRAP antibodies with anti-PvMTRAP antibodies using the same subcellular markers replicated identical fluorescence signal patterns (**Figure 4B**). This confirmed the cross-binding of anti-PvMTRAP to the PkMTRAP antigen and reaffirmed the subcellular localization of PkMTRAP. Pre-immune animal IgG, used as a negative control, did not recognize the parasite antigens (**Supplementary Figure S2**).

### 3.5 Humoral immune responses against PvMTRAP and PkMTRAP

**Figure 5:**
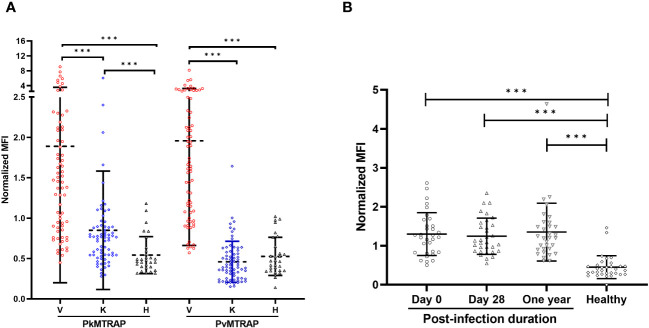
Immunoreactivity of human IgG to PvMTRAP and PkMTRAP. **(A)** Individual IgG responses against PvMTRAP/PkMTRAP in sera from *P. vivax* patients (V, *n*=70), *P. knowlesi* patients (K, *n*=70), or healthy individuals (H, *n*=30). **(B)** IgG responses to PvMTRAP in 32 *P. vivax* patient sera samples collected at different timepoints. The bars indicate the mean and SD; three asterisks indicate *p*<0.001 (Mann–Whitney *U* test).

Recombinant PvMTRAP or PkMTRAP proteins were reacted with sera from *P. vivax* patients (*n*=70), *P. knowlesi* patients (*n*=70) collected from endemic areas, and non-exposed healthy individuals (*n*=30) (**Figure 5A**). The IgG responses of *P. vivax* and *P. knowlesi* patients’ sera to the respective MTRAP antigens were significantly higher than those of non-exposed human sera (*p*<0.001). Furthermore, sera from *P. vivax* patients produced a significant IgG response to PkMTRAP (**Figure 5A**), with an extensive positive correlation with the response to PvMTRAP (*r* ^2 =^ 0.91, 95% CI: 0.85–0.94; **Supplementary Figure S3A**). PvMTRAP induced a high seropositivity of 75.71% (95% CI: 63.99–85.17%), with 96.67% (95% CI: 82.78–99.92%) specificity, in *P. vivax*–infected individuals (**Table 1**). To evaluate the longevity of immune responses, we further conducted the assay with three different sets of sera obtained from *vivax* patients at D0_acute (*n*=32), D28_sub-acute infection (*n*=32), and at least 1 year after infection without reinfection (*n*=32) (**Figure 5B**). The results demonstrated that the IgG responses to PvMTRAP in the sera of *P. vivax* patients were higher than those in the healthy human sera, regardless of the post-infection duration (*p*<0.001). Furthermore, compared to that in acute and sub-acute infection, the IgG response did not significantly change after at least 1 year of infection. Related information on patients with malaria, including age, sex, and parasitemia, is presented in **Supplementary Table S1**. Overall, the protein array data suggest that PvMTRAP is a potential vaccine candidate against *P. vivax*, particularly when coexisting with *P. knowlesi*.

**Table 1:** Seropositivity of IgG response to MTRAP in *P. vivax* and *P. knowlesi* malaria patients determined by protein array.

Antigens	Samples	n	No. of samples		Sensitivity (%)^a^/ Specificity (%)^b^	95% CI ^c^ (%)	Normalized MFI^d^ [Median (IQR)]	*P* value^e^
			Positive	Negative				
PkMTRAP	*P. vivax* exposed	70	46	24	65.71^a^/	53.40 - 76.65	1.45 (0.88 - 2.10)	< 0.001
	*P. knowlesi* exposed	70	13	57	18.57^a^/	10.28 - 29.66	0.76 (0.54 - 0.90)	< 0.001
	Healthy individuals	30	2	28	93.33^b^	77.93 - 99.18	0.47 (0.39 - 0.61)	
PvMTRAP	*P. vivax* exposed	70	53	17	75.71^a^/	63.99 - 85.17	1.65 (1.05 - 2.49)	< 0.001
	*P. knowlesi* exposed	70	2	68	2.86^a^/	0.35 - 9.94	0.39 (0.26 - 0.58)	0.128
	Healthy individuals	30	1	29	96.67^b^	82.78 - 99.92	0.47 (0.35 - 0.68)	

a Sensitivity/seropositive rate: percentage of positive in malaria patient samples.

b Specificity/seronegative rate: percentage of negative in healthy samples.

c Confidence intervals.

d The IgG response is represented by normalized mean fluorescence intensity [MFI]: MFI of the test sample/(MFI + 2 standard deviations of healthy individuals).

e *P* value, the difference in the total IgG response level for each antigen between knowlesi or vivax malaria patients and healthy individuals were calculated with the Mann-Whitney U-test.

### 3.6 Purification of *P. knowlesi* A1-H.1 schizonts using Percoll gradient

**Figure 6:**
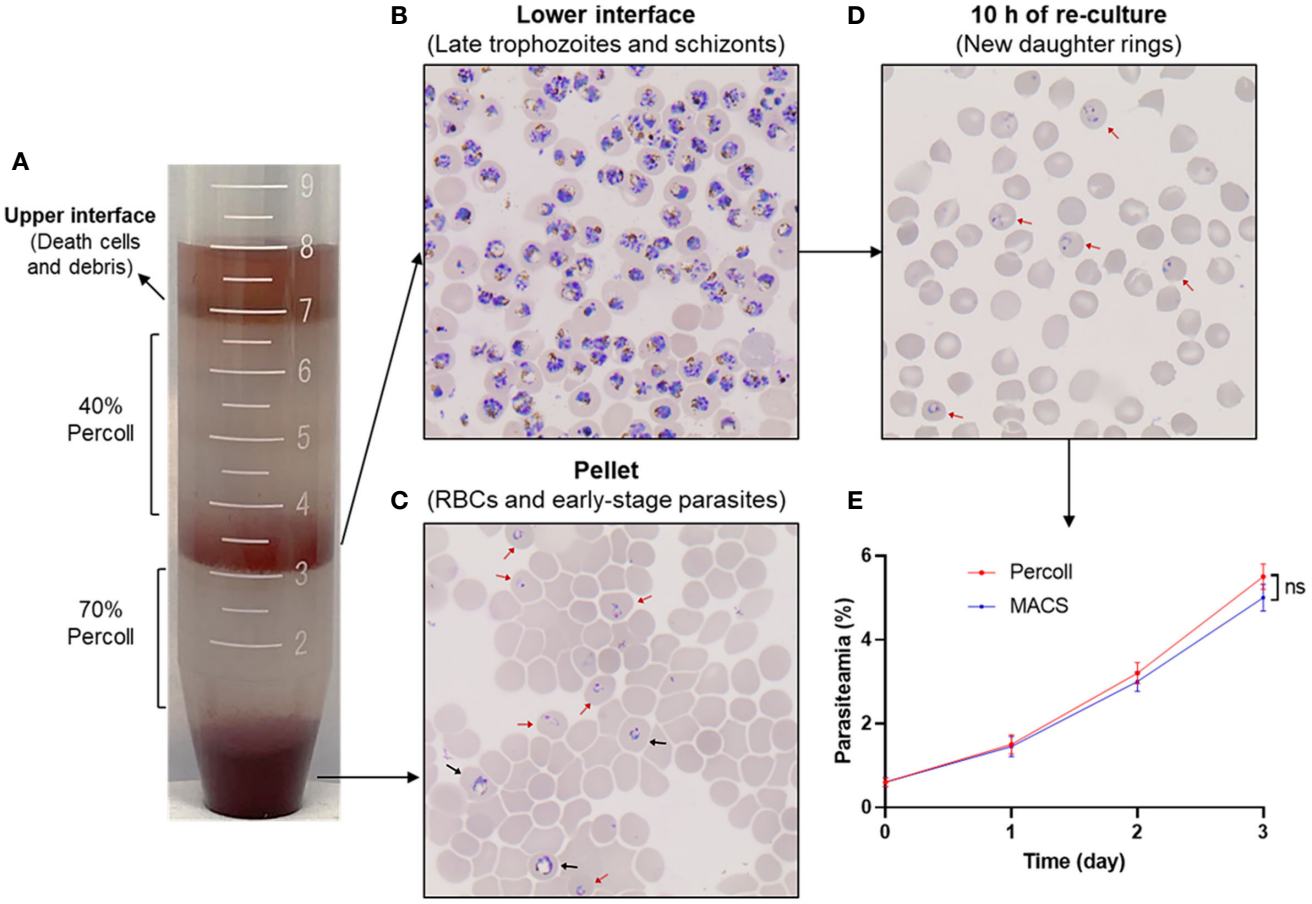
Purification of *P. knowlesi* A1-H.1 schizonts using Percoll gradient. **(A)** A mixture of parasites is overlayed onto a 40%/70% Percoll gradient and fractionated into layers by centrifugation. **(B)** A thin smear of cells from the lower interface layer is stained with Giemsa. **(C)** The cells taken from the pellet are checked after Giemsa staining. **(D)** Cells of the lower interface are re-cultured and examined for development after 10 (h) **(E)** Comparison of the growth rate of culture using enriched schizonts by Percoll gradient and MACS. Red and black arrowheads indicate ring and early trophozoite, respectively.

The enrichment of human erythrocyte-infected *P. knowlesi* A1-H.1 schizont using Percoll gradient is considered a reasonable method, based on successful experiments on other strains of *P. knowlesi* maintained with either monkey or human red blood cells (Ginsburg et al., 1987; Moraes Barros et al., 2017; Armistead et al., 2018). Various concentrations of Percoll were assessed before choosing the 70% cushion for efficiently purifying late-stage parasites. Additionally, a 40% Percoll cushion effectively eliminated dead cells and debris (data not shown). Using the 40%/70% Percoll gradient (**Figure 6A**), a majority of late-stage parasites were concentrated within the lower interface layer (**Figure 6B**), thereby effectively separating most of the rings, early trophozoites, and uninfected human erythrocytes in the pellet (**Figure 6C**). The enriched late-stage parasites were re-cultured, and subsequently developed into new daughter rings within approximately 10 h (**Figure 6D**), but without significantly impacting parasite growth (**Figure 6E**). This confirmed that the 40%/70% Percoll gradient is appropriate for preparing *P. knowlesi* A1-H.1 schizonts for downstream invasion inhibition assays.

### 3.7 Parasite invasion inhibitory ability

**Figure 7:**
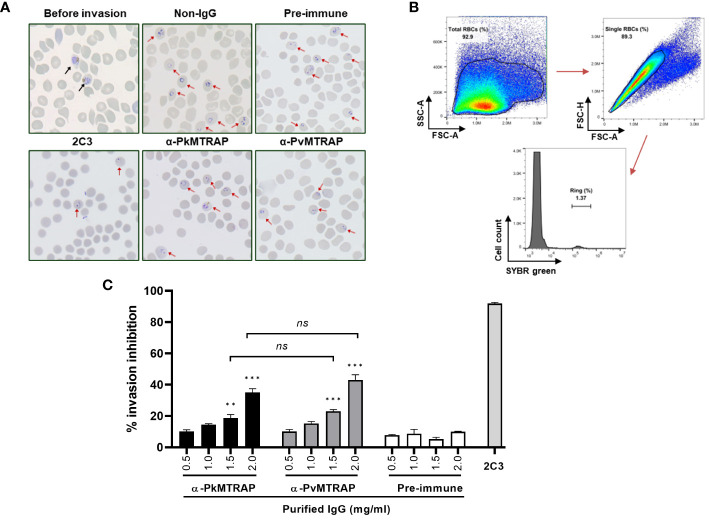
Inhibition of *P. knowlesi* invasion by antibodies against PkMTRAP and PvMTRAP. **(A)** Thin smear with Giemsa staining for parasites at the starting point (before invasion) and within 10 h after incubation. Different conditions are examined, including untreated schizonts (non-IgG) or schizonts cultured with pre-immune IgG, purified IgG against PvMTRAP (α-PkMTRAP) or PkMTRAP (α-PkMTRAP), with anti-DARC (2C3) as a positive control. **(B)** Gating strategies of flow cytometric data to calculate the percentage of the ring form. **(C)** Invasion-inhibitory activity of antibodies evaluated by flow cytometry. Data are presented as percentages (mean and SEM) of invasion inhibition from three biological replicates. Significant differences are indicated by ** (*p*<0.01) and *** (*p*<0.001) (Student’s *t*-test). ns, not significant.

A growing body of practical evidence supports the use of *in vitro* culture *P. knowlesi*-based invasion inhibition assays for screening potential asexual blood-stage vaccine candidates from *P. vivax* (Muh et al., 2018a; Han et al., 2019; Muh et al., 2020; Ndegwa et al., 2021). In the present study, we employed the previously established method using SYBR Green I as a specific DNA staining to evaluate the ability of purified rabbit IgG against PkMTRAP or PvMTRAP to prevent the parasite from invading erythrocytes (Muh et al., 2018b). The healthy ring-form morphology of the parasite was obtained from schizonts cultured without any intervention as well as antibody-treated schizonts, suggesting that the function of antibodies (if extant) was related to the erythrocyte invasion rather than to other steps of the schizont-to-ring stage development (**Figure 7A**). The percentage of daughter rings was evaluated by flow cytometry using manual gating strategies (**Figure 7B**). Both purified anti-PkMTRAP and anti-PvMTRAP rabbit IgG suppressed the erythrocyte invasion of *P. knowlesi* parasites in a concentration-dependent manner. At IgG concentration ≥1.5 mg/mL, the anti-PkMTRAP and anti-PvMTRAP antibody invasion inhibition activity was significantly higher than that of pre-immune IgG. Within the tested range, the best inhibitory performance of antibodies against PkMTRAP and PvMTRAP was 35.02 ± 2.48% and 43.08 ± 3.28%, respectively, without significant difference between the two antibodies (**Figure 7C**).

## 4 Discussion

It is well-documented that proteins of the TRAP family considerably contribute to the most important parts of the malarial parasite’s life cycle in both human and mosquito hosts. For instance, TRAP is crucial for the invasion of the mosquito salivary glands and mammalian liver cells of sporozoites (Sultan et al., 1997); moreover, TREP is essential for sporozoite gliding motility and invasion of salivary glands (Combe et al., 2009; Steinbuechel and Matuschewski, 2009) whereas CTRP has a role in the invasion of the mosquito midgut (Dessens et al., 1999). Due to specific expression in blood-stage merozoites and conserved TSR structures related to parasite adhesion, MTRAP has been assumed to play a role in the erythrocytic stage (Morahan et al., 2009). For instance, MTRAP was a prerequisite for *P. falciparum* egression from the erythrocyte, as the parasite failed to disrupt the parasitophorous vacuole membrane without MTRAP’s contribution (Bargieri et al., 2016). MTRAPs from *P. falciparum* and other primate-infected *Plasmodium* spp. have been intensively reported; however, to our knowledge, this research is the first to describe the characterizations of MTRAP from non-*P. falciparum* human malaria parasites, specifically in the two phylogenetically close species, *P. vivax* and *P. knowlesi*.

Despite countless efforts, *P. vivax* could not be cultured *in vitro* for a long time, which hindered the progress in the development of vaccines and therapeutics against this pathogen. Fortunately, *P. knowlesi*, which is closely related phylogenetically to *P. vivax*, can be maintained for prolonged periods *in vitro* with human erythrocytes and has been utilized as a model for studying *P. vivax* in accumulated reports (Muh et al., 2018a; Han et al., 2019; Verzier et al., 2019; Muh et al., 2020; Ndegwa et al., 2021). Ndegwa and colleagues (Ndegwa et al., 2021) successfully produced chimeric *P. knowlesi* lines by exchanging Pk41, Pk12, PkARP, and PkGAMA with their *P. vivax* orthologs, and then utilized these parasite lines in downstream immunofluorescence and invasion inhibition assays. The fluorescence signals thus obtained exhibited identical patterns to those observed in wild-type *P. knowlesi*; this offered strong evidence that the tested *P. vivax* proteins and their orthologs share the same localization within the merozoite invasion organelle. In this regard, the subcellular localization of PvMTRAP may be identical to that of PkMTRAP, likely the rhoptry body; however, an assay using immunoelectron microscopy with *P. knowlesi* and *P. vivax* (if possible) parasites is indispensable for further confirmation. Furthermore, by localizing to distinct invasive organelles of merozoites, PvMTRAP and PkMTRAP may have different functional roles compared to those of PfMTRAP in the development of the respective parasites.

The inhibitory effect of rabbit IgG against *P. vivax* proteins on erythrocyte invasion through transgenic *P. knowlesi* lines expressing respective *P. vivax* antigens was comparable to that of the wild-type *P. knowlesi* (Ndegwa et al., 2021). Given the numerous *P. vivax* antigens that still lack characterization (Siau et al., 2023) and the labor-intensive, time-consuming nature of gene editing, wild-type *P. knowlesi* could be useful in screening *P. vivax* proteins using an invasion inhibition assay. For the assay’s initial step, mature forms of parasitized erythrocytes were enriched from a mix of early-stage parasites and uninfected erythrocytes. This crucial step mostly relied on the buoyant density differences between naïve RBCs and developmental stages of infected RBCs or the paramagnetic properties of hemozoin present in late-stage parasites (Dluzewski et al., 1984; Moon et al., 2013; Amir et al., 2016). Due to low viscosity, separation using Percoll requires lower centrifugal forces and a shorter experimental duration that might induce greater integrity of the parasites and host cells (Tosta et al., 1980). Regarding the changing density of parasitized erythrocytes according to the maturation of the parasite, we considered that the divergent parasite strains and sources of host cells might result in a different optimal condition of Percoll gradient centrifugation (Tosta et al., 1980; Dluzewski et al., 1984; Reed et al., 2000; Moraes Barros et al., 2017; Ong et al., 2023; Siau et al., 2023); however, reports on *P. knowlesi* A1-H.1 infecting human erythrocytes are lacking. In this context, we examined various concentrations of Percoll and found that the 40%/70% gradient yielded the best results in terms of high purity and viability of *P. knowlesi* A1-H.1 schizonts, which were crucial for the downstream invasion inhibition assay (**Figure 6**). In our study, we used *P. knowlesi* A1-H.1 parasites in combination with polyclonal antibodies against PvMTRAP and PkMTRAP for the invasion inhibition assay. The rabbit IgGs (2.0 mg/mL) against PkMTRAP or PvMTRAP reduced parasite invasion into erythrocytes by approximately 40% compared to the control (**Figure 7C**). The activity of these antibodies increased in a concentration-dependent manner, indicating specific inhibition. This result was notable because the antibody against PfMTRAP did not prevent *P. falciparum* parasites from invading erythrocytes, even with the specific receptor on the erythrocyte present (Bartholdson et al., 2012). Initially, attempts to knock out the *pfmtrap* gene resulted in failure, which posed a challenge in confirming the function of this protein during the blood stage of *P. falciparum* (Baum et al., 2006). However, using CRISPR-Cas9 technology, Bargieri et al. successfully disrupted *mtrap* in *P. falciparum*, demonstrating that MTRAP is not essential for the asexual blood stage of *P. falciparum* (Bargieri et al., 2016). This discovery strongly corroborated previous invasion inhibition data, revealing that *P. falciparum* merozoites can invade human erythrocytes in the absence of PfMTRAP protein. The diverse functionality of MTRAPs might be attributed to differences in the protein sequence and/or subcellular localization of PfMTRAP, as compared to PvMTRAP and PkMTRAP, a revelation of this study. Furthermore, our recent study demonstrated that PvMTRAP specifically interacts with human CD36, a reticulocyte surface marker (Nguyen et al., 2023); this interaction may differ from that between PfMTRAP and human CD108, which appears unrelated to erythrocyte invasion. Future research is crucial to investigate the presence and role of MTRAPs in the asexual blood stage and, more broadly, the roles of other TRAP family members of *P. vivax* and *P. knowlesi* as potential vaccine and therapeutic targets.

Our protein array data indicated that, regarding antigenicity, PvMTRAP represents a promising target for vaccine development. This apical organelle protein elicited a human immune response in 75.7% of tested *P. vivax* patients (**Table 1**), comparable to that of PvMSP1–19 (79.2%) but notably higher than that of other merozoite surface proteins like Pv41 (62.5%), PvMAS180 (44.4%), and apical organelle proteins, for instance, PvRAMA (63.5%) and PvAARP (50.0%), as determined using the same methodology previously (Cheng et al., 2013; Lu et al., 2014; Muh et al., 2018a). Furthermore, there was sustained immunoreactivity against PvMTRAP for at least 1 year after *P. vivax* infection without re-infection or recrudescence. The percent sequence identity of proteins from *P. vivax* and their orthologs in *P. knowlesi* correlated with their cross-reactivity level (Longley et al., 2022). As expected, cross-reactive immune responses to PkMTRAP occurred in 65.7% (95% CI, 53.4%–76.7%) of sera samples from *P. vivax* patients (**Table 1**). This is in line with full-length proteins share 78.0% identity, and their TSR domains were even more conserved in sequence and predicted 3D structures. Nonetheless, an incomparable IgG response of the sera of patients with *P. knowlesi* to PkMTRAP and PvMTRAP suggested that humoral immunogenicity against PkMTRAP might be partially obscured by competitive antigen(s) from *P. knowlesi* (Kallas et al., 2019). Furthermore, a previous screening study identified three distinct types of immunoreactivities between IgG from patients with vivax and/or knowlesi malaria and the *P. vivax* and/or *P. knowlesi* antigens. Accordingly, the IgG response to MTRAP was categorized among those where the sera of patients with *P. vivax* infection are more reactive to *P. knowlesi* antigens than is the sera of patients with *P. knowlesi* (Muh et al., 2020). *Plasmodium knowlesi* and *P. vivax* have been co-endemic across most Southeast Asian regions (Anstey and Grigg, 2019). Thus, vaccine candidates covering both of these species based on the specific response to a corresponding species and cross-reactivity to the other concurrently should be prioritized. In this regard, PvMTRAP may be an attractive option for developing a vaccine against vivax and knowlesi malaria.

In summary, PkMTRAP and PvMTRAP were localized on the rhoptry body of merozoites in blood-stage parasites, and both were capable of eliciting immune responses in patients from endemic areas. The immune response to PvMTRAP demonstrated high immunogenicity, longevity, and cross-reactivity with *P. knowlesi*. The antibody against PvMTRAP may be as effective as the anti-PkMTRAP antibody for inhibiting erythrocyte invasion by *P. knowlesi*. This suggests that MTRAP is a potential target for the development of novel drugs and vaccines against vivax and knowlesi malaria.

## Data availability statement

The original contributions presented in the study are included in the article/**Supplementary Material**. Further inquiries can be directed to the corresponding author.

## Ethics statement

All experiments were obtained in accordance with relevant guidelines and regulations and all experimental protocols involving human samples were approved by the Institutional Ethical Committee of the Kangwon National University Hospital Ethical Committee (IRB No. KNUH-B-2021-06-034), Department of Medical Research, Myanmar (Approval number 49/Ethics-2014), University of Malaysia Medical Ethics Committee (Ref No. 817.18) and the Medical Research Ethics Committee (MREC), Ministry of Health, Malaysia (National Medical Research Register ID No. 13079). The studies were conducted in accordance with the local legislation and institutional requirements. The human samples used in this study were acquired from primarily isolated as part of your previous study for which ethical approval was obtained. Written informed consent for participation was not required from the participants or the participants’ legal guardians/next of kin in accordance with the national legislation and institutional requirements. The animal study was reviewed and approved by the Institutional Animal Care and Use Committee of Kangwon National University, and the experiments were conducted according to the Ethical Guidelines for Animal Experiments of Kangwon National University (KW-220620-3). The study was conducted in accordance with the local legislation and institutional requirements.

## Author contributions

NS: Data curation, Formal analysis, Investigation, Methodology, Software, Visualization, Writing – original draft, Writing – review & editing. T-KN: Data curation, Formal analysis, Investigation, Methodology, Resources, Writing – review & editing. NT: Investigation, Methodology, Resources, Writing – review & editing. T-TC: Investigation, Methodology, Writing – review & editing. S-HN: Resources, Writing – review & editing. RM: Methodology, Resources, Supervision, Writing – review & editing. YL: Methodology, Resources, Writing – review & editing. MHN: Methodology, Resources, Writing – review & editing. W-SP: Methodology, Validation, Writing – review & editing. W-JC: Methodology, Validation, Writing – review & editing. FL: Methodology, Resources, Validation, Writing – review & editing. S-KL: Data curation, Formal analysis, Methodology, Writing – review & editing. J-HH: Data curation, Methodology, Resources, Validation, Writing – review & editing. E-TH: Conceptualization, Funding acquisition, Project administration, Supervision, Validation, Writing – original draft, Writing – review & editing.
