# Supplementary material for: Characterization of merozoite-specific thrombospondin-related anonymous protein (MTRAP) in Plasmodium vivax and P. knowlesi parasites
Source: Front Cell Infect Microbiol. 2024 Feb 23;14:1354880. doi: 10.3389/fcimb.2024.1354880 (PMC10920329; doi:10.3389/fcimb.2024.1354880)
Supplement: Supplementary file 1 [file DataSheet_1.docx]

***Supplementary Materials***

**Characterization of merozoite-specific thrombospondin-related anonymous protein (MTRAP) in *Plasmodium vivax* and *P. knowlesi* parasites**

Nguyen Sy Thau^1^, Tuyet-Kha Nguyen^1^, Truong Van Nguyen^1^, Thanh-Hang Chu-Thi^1^, Sung-Hun Na^2^, Robert W. Moon^3^, Yee Ling Lau^4^, Nyunt Myat Htut^5^, Won-Sun Park^6^, Wan-Joo Chun^7^, Feng Lu^8^, Seong-Kyun Lee^1^, Jin-Hee Han^1^, Eun-Taek Han^1^*

*** Corresponding:** Eun-Taek Han: ethan@kangwon.ac.kr; etaekhan@gmail.com

**1. Supplementary Figures**

| 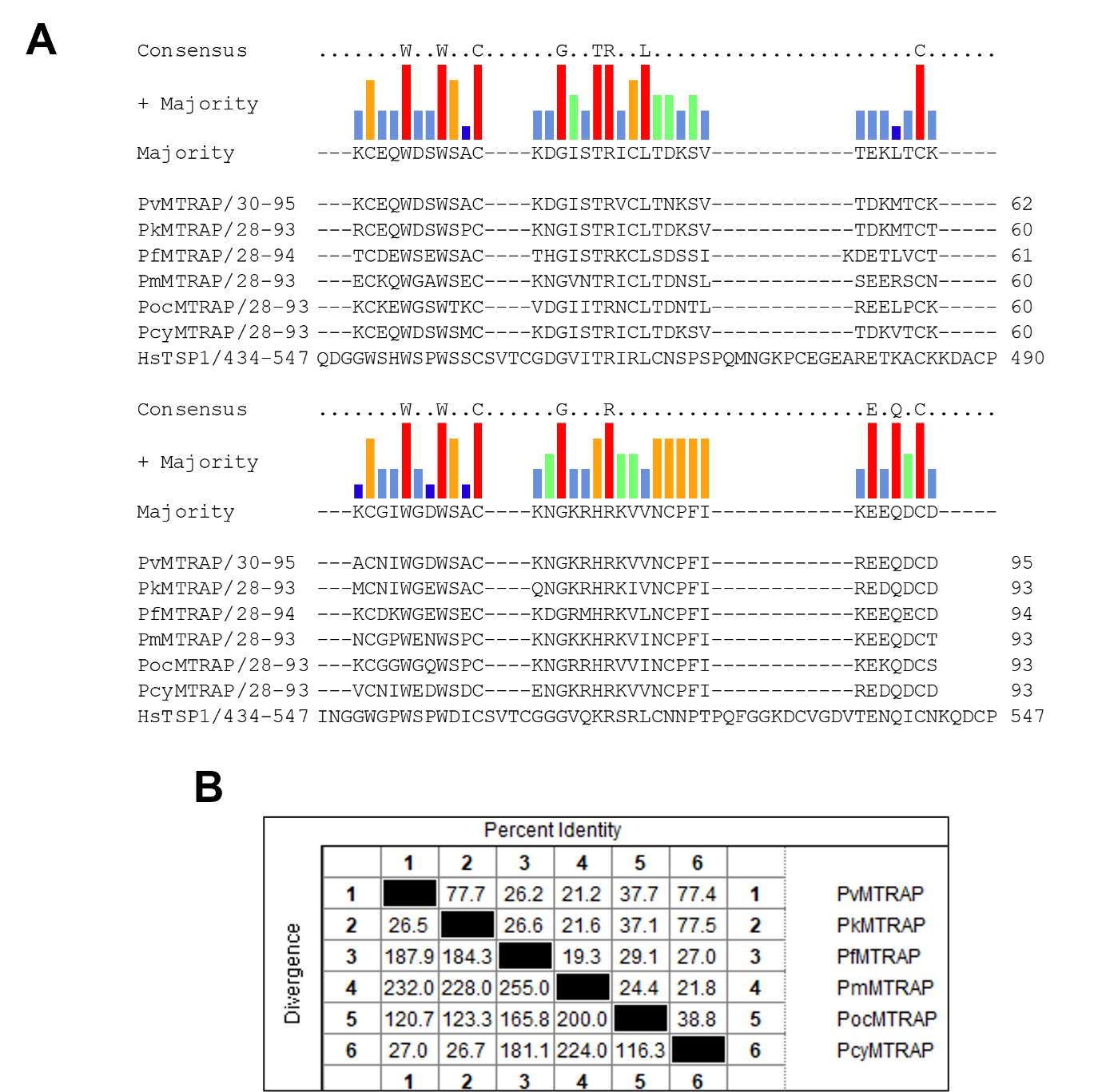 |  |
| --- | --- |
| **Supplementary Figure 1.** Sequence analysis of MTRAP proteins of human malaria parasites. **(A)** Multiple sequence alignment of MTRAP TSR domains of human-infected *Plasmodium* with human thrombospodin-1 protein (HsTSP-1). There are repeated conserved sequences of ‘WxxWxxC~G~R~C’ (‘x’ could be substituted by any amino acid). **(B)** Percent identity and divergence (in percent) of full-length MTRAP proteins of human malaria parasites. |  |
| 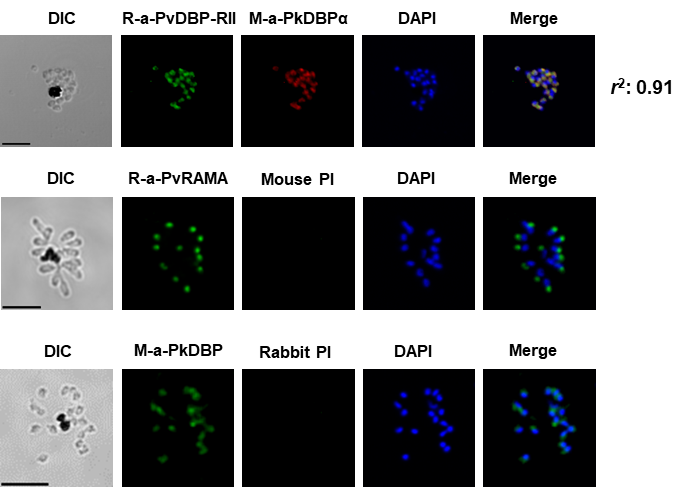 | |
| **Supplementary Figure 2.** Control antibodies for immunofluorescent assay. The signal of antibodies against PvDBP-RII and PkDBPα with calculated Pearson Coefficient of *r*^2^=0.91 as a control for overlapping signal (the top row). The IgG from pre-immune mouse (the middle row) and pre-immune rabbit (the bottom row) did not show any fluorescence signal. | |

| 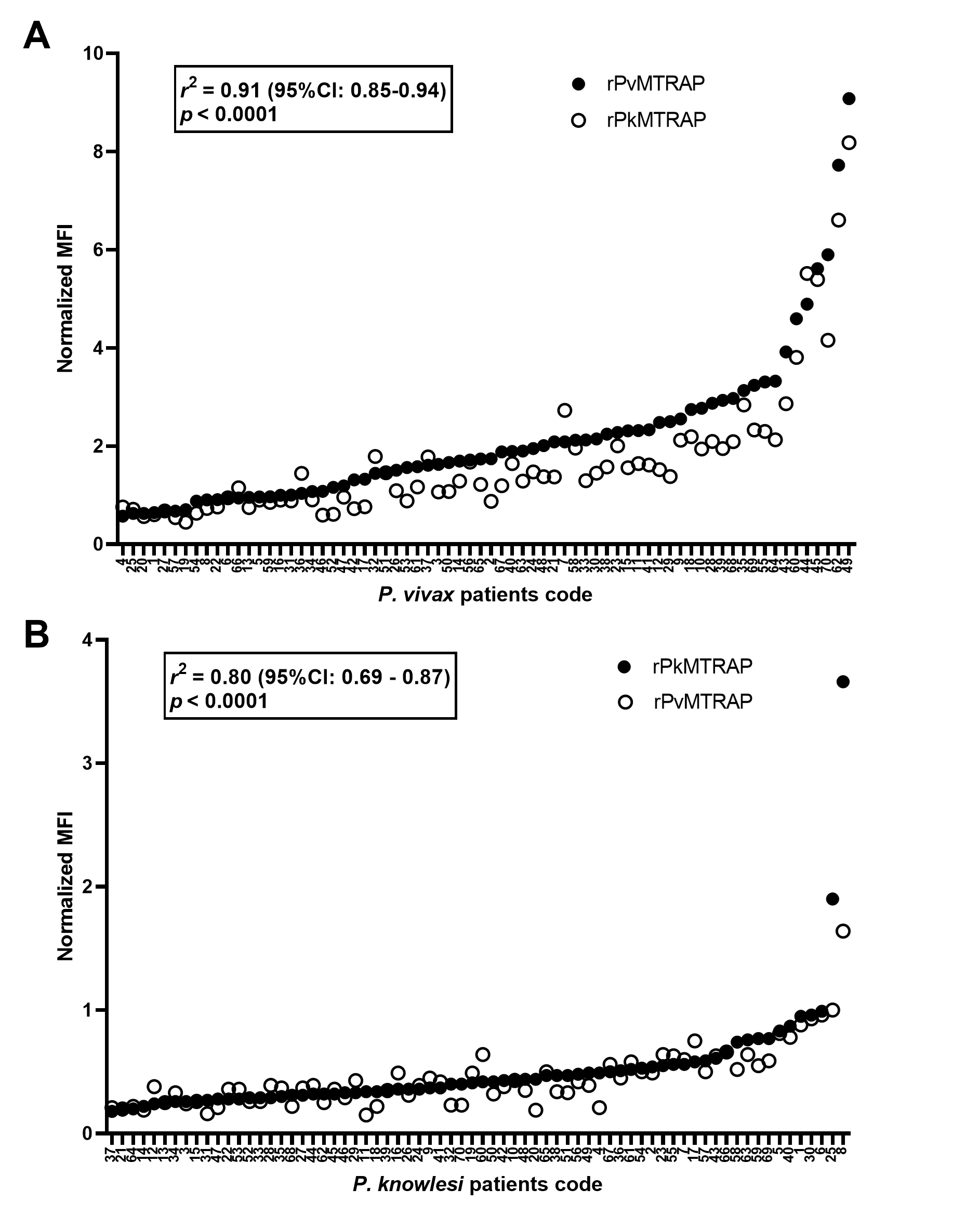 |
| --- |
| **Supplementary Figure 3.** Correlation analysis of malaria patients IgG response to PvMTRAP and PkMTRAP detected by protein array. **(A)** IgG response level of *P. vivax* patients sera against recombinant PvMTRAP (rPvMTRAP, solid dot) and rPkMTRAP (non-filled dot). **(B)** IgG response level of *P. knowlesi* patients sera against rPkMTRAP (solid dot) and rPvMTRAP (non-filled dot). The *r*^2^ and *P*-value were produced by using a nonparametric Spearman correlation analysis. |

2. **Supplementary Table**

| \|  \| **Korean *P. vivax* patients** \| **Myanmar *P. vivax* patients** \| ***P. knowlesi* patients** \| \| --- \| --- \| --- \| --- \| \| **Total (n)** \| 70 \| 32 \| 70 \| \| **Gender (% of male)** \| 79.24 \| 87.50 \| 73.33 \| \| **Age (year)** \| \| \| \| \| Mean (SD) \| 24.16 (12.53) \| 21.00 (7.14) \| 36.95 (18.10) \| \| Range \| 5 - 74 \| 18 - 23 \| 6 - 72 \| \| **Parasitemia (%)** \| \| \| \| \| Median \| 0.07 \| 0.40 \| 0.70 \| \| 25% - 75% IQR \| 0.03 – 0.15 \| 0.23 - 0.60 \| 0.28 – 2.10 \| |
| --- | --- | --- | --- | --- | --- | --- | --- | --- | --- | --- | --- | --- | --- | --- | --- | --- | --- | --- | --- | --- | --- | --- | --- | --- | --- | --- | --- | --- | --- | --- | --- | --- | --- | --- | --- | --- |
| **Supplementary Table 1.** Baseline information of malaria patients contributed sera using in protein array. |
